# Neural alignment during outgroup intervention predicts future change of affect towards outgroup

**DOI:** 10.1093/cercor/bhae125

**Published:** 2024-04-02

**Authors:** Annika Kluge, Niko Somila, Kaisu Lankinen, Jonathan Levy

**Affiliations:** Department of Neuroscience and Biomedical Engineering, Aalto University, Espoo 02150, Finland; Department of Neuroscience and Biomedical Engineering, Aalto University, Espoo 02150, Finland; Athinoula A. Martinos Center for Biomedical Imaging, Department of Radiology, Massachusetts General Hospital, Boston, MA 02114, USA; Department of Radiology, Harvard Medical School, Boston, MA 02115, USA; Department of Neuroscience and Biomedical Engineering, Aalto University, Espoo 02150, Finland; Department of Criminology and Gonda Multidisciplinary Brain Research Center, Bar-Ilan University, Ramat Gan 5290002, Israel

**Keywords:** intergroup bias, magnetoencephalography, multiset canonical correlation analysis, paradoxical thinking, vaccination attitudes

## Abstract

While social psychology studies have shown that paradoxical thinking intervention has a moderating effect on negative attitudes toward members from rival social groups (i.e. outgroup), the neural underpinnings of the intervention have not been studied. Here, we investigate this by examining neural alignment across individuals at different phases during the intervention regarding Covid-19 vaccine-supporters’ attitudes against vaccine-opposers. We raise two questions: Whether neural alignment varies during the intervention, and whether it predicts a change in outgroup attitudes measured via a survey 2 days after the intervention and compared to baseline. We test the neural alignment using magnetoencephalography-recorded neural oscillations and multiset canonical correlation analysis. We find a build-up of neural alignment which emerges at the final phase of the paradoxical thinking intervention in the precuneus—a hub of mentalizing; there was no such effect in the control conditions. In parallel, we find a behavioral build-up of dissent to the interventional stimuli. These neural and behavioral patterns predict a prosocial future change in affect and actions toward the outgroup. Together, these findings reveal a new operational pattern of mentalizing on the outgroup, which can change the way individuals may feel and behave toward members of that outgroup.

## Introduction

There is an alarming uptick in violent conflicts around the world (Roser et al. 2016). Conflict perpetuating factors include negative attitudes against rivaling social groups (i.e. outgroups): prejudice, intergroup bias, and even support for violence (Hameiri et al. 2014a; Saguy and Reifen-Tagar 2022). Thereby, social psychologists have been developing and testing interventions to moderate negative outgroup attitudes (Bar and Hameiri 2020; Paluck et al. 2021).

A recent review studied prejudice reducing interventions from the last decade and concluded that the long-term impact of the interventions is unclear, and few methodologically sound studies find substantial effects (Paluck et al. 2021). Still, this conclusion is based on short-term self-reports which do not predict real-life behavior well (Kurdi et al. 2019): the review found that only 8% of the studies test the intervention effects even a day later (Paluck et al. 2021). Lately, more intergroup intervention studies have turned to neuroimaging to find attitude change predictors (Hautala et al. 2022; Levy et al. 2022), but still search for affected neural mechanisms after the intervention, overlooking the mechanisms operating during the intervention itself. A few exceptions from other cognitive fields have shown good results in predicting real-life outcomes (Falk et al. 2010, 2012; Berkman and Falk 2013; Kang and Falk 2020), proposing the potential of this approach in the field of intergroup interventions.

Coming to specific outgroup interventions, paradoxical thinking uses consistent information to the persons’ beliefs but takes it to an exaggerated level, evoking varying levels of agreement and unfreezing polarizing attitudes ( Hameiri et al. 2014b; Hameiri et al. 2016; Bar et al. 2021; Knab and Steffens 2022). It may lead to perceiving one’s own group attitudes as irrational and questioning the group identity as a whole (Hameiri et al. 2018), therefore reducing polarization ( Hameiri et al. 2014b). The intervention has shown to be more effective in moderating negative attitudes than exposing participants to extremely inconsistent information, which is known to not be effective in highly hostile intergroup situations and can be used as a control for paradoxical thinking (Hameiri et al. 2018; Bar and Hameiri 2020; Hautala et al. 2022). The neural mechanisms activating during the paradoxical thinking intervention itself, however, have not yet been directly tested and it is not clear whether the neural change would be instant or have a build-up.

Here we look at the group dynamics between the vaccinated and unvaccinated participants against Covid-19, group identities that rapidly became prominent (Henkel et al. 2022) and resulted in affective polarization (Bajwa 2021; Jiang et al. 2021). Our recent study found that paradoxical thinking impacts the vaccination-related attitudes (Hautala et al. 2022). In addition, we recently demonstrated that examining neural oscillations measured using magnetoencephalography (MEG) proves useful in revealing new insights into intergroup affect (Levy et al. 2016; Levy and Feldman 2017), attitudes ( Levy et al. 2021a), and behavior change (Hautala et al. 2022; Levy et al. 2022) even in a pre-post design, specifically helping us understand which neural mechanisms are modulated as a consequence of the interventions. In this paper, we aim to broaden that insight by looking at the neural oscillations recorded during the intervention itself and exploring *how* the intervention changes the neural mechanisms.

For this reason, our goal is to uncover consistent brain signals across participants and across time in response to the intervention: naturalistic stimuli concerning outgroup attitudes. Many studies have used functional magnetic resonance imaging (fMRI) to study neural alignment (e.g. signal similarity across participants; Hasson et al. 2004; Jääskeläinen et al. 2008; Lahnakoski et al. 2012; Nummenmaa et al. 2012; Shaw et al. 2018) but there is an alternative for MEG data with better temporal resolution while recording rhythmic activity behind the responses to naturalistic stimuli. MEG bypasses the intermediate processes of neurovascular coupling of fMRI and the signal distortion due to the scalp of electroencephalography (EEG; Levy et al. 2021b). Multi-set canonical correlation analysis (MCCA; Kettenring 1971; Yi-Ou Li et al. 2009) is an analysis method to find consistencies across participants which operates on the whole brain level (Lankinen et al. 2018) and is especially valid in naturalistic situations (Lankinen et al. 2014; Levy et al. 2021b).

To summarize, in this study, we used MCCA on MEG data recorded while participants went through the paradoxical thinking intervention in the context of Covid-19 vaccines and sought (hypothesis 1) whether neural alignment varies during paradoxical manipulation. We also test this in two control conditions: inconsistency approach (as a balanced reverse approach to paradoxical thinking) and neutral (unrelated to vaccination) control. Additionally, we examine (hypothesis 2) whether the neural alignment predicts change in self-reported outgroup attitudes not right after the intervention, but a couple of days later to examine whether the effect sinks in.

## Materials and methods

### Experimental design

We tested vaccine-supporters who were at least moderately negative against vaccine-opposers (mean 5.00, 95% CI [4.85, 5.14] on a scale of 1–7, where 7 reflects extremely negative and 1 reflects extremely positive attitudes) as reported via an online questionnaire (filled on average 2 weeks before the intervention). One hundred and twenty-one (121) healthy (no self-reported acute neurological illnesses or psychiatric disorders) adults participated in the experiment. One participant’s neural data was not recorded during the intervention; they were excluded from further analysis. Our final pool was 120 participants (native Finns, 61.7% female, age ranging from 18.9 to 57.5 years with a median at 24.3) randomly divided into paradoxical (*n* = 39), inconsistent (*n* = 40), and control (*n* = 41) group, controlling for gender and negativity toward vaccine-opposers. They listened to 22 auditory statements while neural data were recorded by MEG. We measured their explicit attitudes again after the intervention (on average, 1.7 days after the MEG measurement). This data collection was preregistered (https://osf.io/uwmpa/?view_only=e48e1c57ad8f4639ba35a974b92122aa). The current hypotheses and analysis plan were not preregistered, as the current study is complimentary to the preregistered analysis and focuses on the intervention mechanisms. As such, this paper uses the explicit self-reports from before and after the intervention, and the neural data and self-reported agreements recorded during the auditory intervention.

All participants read an information sheet and a privacy notice paper and signed the participation confirmation form, approved by the Aalto University Research Ethics Committee. All experiments were performed in accordance with the instructions by the Finnish Advisory Board on Research Integrity and General Data Protection Regulation (GDPR).

### Explicit measures

**Fig. 1:**
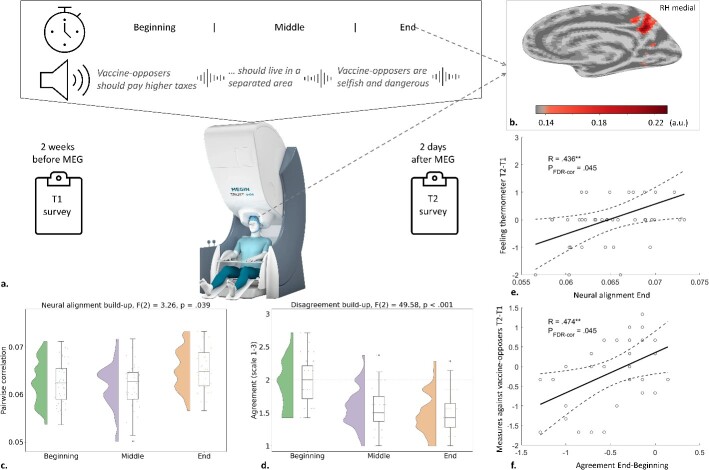
Experiment and results for the paradoxical thinking group. (a) Experimental design. Participants sat in MEG device (device picture rights MEGIN; https://megin.com/), while listening to interventional statements. We split the experimental data into three timeslots. Participants filled the pre-questionnaire (T1) 2 weeks before the MEG measurement and the post-questionnaire (T2) 2 days after. (b) The end-timeslot of delta frequency range in the paradoxical group produced a significant inter-subject activation in precuneus. (c) The neural alignment grew during the intervention in the delta frequency range of the paradoxical thinking group. (d) The agreement with extremely pro-vaccination statements reduced during intervention for the paradoxical thinking group. (e) The end-timeslot intersubject correlation in delta frequency range of the paradoxical thinking group predicted change in outgroup affect. (f) The change in agreement predicted change in support for measures against vaccine-opposers for the paradoxical thinking group.

Before and after the experiment, all participants filled out an online questionnaire (Fig. 1a). The scales investigated were used in both (pre and post) questionnaires and are the following: negativity against vaccine hesitancy (7 items, some reverse-scored, on a scale of 1–7, 1 being “totally disagree”, 7 being “totally agree”); perceived threat (4 items, on a scale of 1–7, 1 being “totally disagree,” 7 being “totally agree”); feeling thermometer toward vaccine hesitancy (on a scale of 1–10, 1 being very cold feelings, 10 being very warm feelings); dehumanization of vaccine hesitant individuals (on a scale of 1–10, 1 being not at all human, 10 being very much human); perceived competence and warmth of vaccine-hesitant individuals (4 items in both categories, on a scale of 1–5, 1 being “not at all,” 5 being “very much”); support for measures against vaccine-opposers (3 items, on a scale of 1–7, 1 being “not at all,” 7 being “very much”); emotions about vaccine hesitancy (8 items separately, on a scale of 1–7, 1 being “not at all’, 7 being ‘very much”).

For this last scale of emotions, we combined individual emotions into two variables: positive (empathy, sympathy, compassion) and negative (anger, hate, fear, shame, guilt) affect. The participants responded to the pre-questionnaire 2 weeks before (to be precise, 13.37 days, 95% CI [6.06, 20.67]) and to the post-questionnaire 2 days (to be precise, 1.70 days, 95% CI [0.56, 2.83]) after the MEG measurement on average.

### Intervention

All manipulations consisted of 22 auditory statements. In the paradoxical thinking group participants listened to exaggerated vaccine-supporting statements (Hameiri et al. 2014b; Knab and Steffens 2022), for example, “Vaccine-opposers should live in a separated area”. Participants assigned to the inconsistent group listened to vaccine-opposing statements (Bartunek 1993), for example, “Vaccines weaken the immune system”. In the control group, participants were exposed to neutral, unrelated statements, for example, “During pregnancy, one should avoid smoking”. All statements are reported in OSF: https://osf.io/8tgc3. The paradigm lasted about 9 minutes and was presented via a Panphonics SoundShower speaker and Presentation software (Neurobehavioral Systems Inc.; version 22.0, Berkeley, CA, USA). The statements were 11–18 s long. After each statement, participants rated how much they agree with the message on a scale of 1 (disagree) to 3 (agree). The order of the statements was fixed for MCCA validity—the method requires all participants to listen to the exact same stimuli at each time instant to look for neural synchrony.

### Neural measures

During the intervention, the participants were seated in a magnetically shielded room in the MEG Core of Aalto Neuroimaging infrastructure at Aalto University and their continuous rhythmic neural activity was recorded with a 306-channel neuro-magnetometer (VectorView, Elekta-Neuromag, Helsinki, Finland). We analyzed neural data from only the time participants listened to statements, removing the pauses.

### MCCA procedure

Our goal was to uncover consistent brain signals across participants (Lankinen et al. 2014), and compare them at different timepoints of the intervention. We used MCCA (Kettenring 1971; Yi-Ou Li et al. 2009) with spatial filtering of MEG data to find the brain signals with the strongest correlation between participants. This approach applied into MEG data is described in more detail in Lankinen et al. 2014 and Lankinen et al. 2016. We performed analyses in three functional frequency bands: delta (0.5–4 Hz), theta (4–8 Hz), and alpha (8–12 Hz) (Buzsáki 2006) with the main focus on the delta band based on earlier research (Lankinen et al. 2014, 2018). We divided each intervention group (paradoxical, inconsistent, control) into three timeslots: beginning, middle, and end part of the intervention, each part consisting of approximately 2 minutes, optimizing between looking at trends across timeslots and the robustness of the analysis of each timeslot (Fig. 1a). We analyzed each experimental group, frequency band and timeslot separately and identically (hereby named “each group” for short). We used 3-fold cross-validation for model training and testing in each group and chose the MCCA component with the strongest correlation between the participants for source analysis and the comparison across timeslots. Sensor-level MCCA analysis was performed using MATLAB 2023b ( MATLAB 2022) and Fieldtrip (Oostenveld et al. 2011). For source analysis, we first calculated a forward model, covariance matrix and inverse model for each participant. We used *fsaverage* from Freesurfer (Fischl 2012) to calculate the forward models with default parameters. Then, we projected the individual sensor activation patterns to source space by applying an inverse model to the activation pattern vector. Finally, we averaged the source estimates of activation across participants and morphed them on an average head. Source analysis was conducted with MNE-python (Gramfort 2013). Analysis code is available at https://version.aalto.fi/gitlab/klugea1/mcca_statements/. We used Rainclouds for plotting (Allen et al. 2021) and Talairach client to find source labels (Lancaster et al. 1997, 2000).

### Statistical analysis

We tested the statistical significance of the selected MCCA component by nonparametric circular bootstrapping, estimating the p-values for the correlation coefficients from the null distribution, similarly to previous studies (Lankinen et al. 2014, 2016). We compared the strongest components’ intersubject correlation (ISC) of the three timeslots between intervention groups in each frequency band using a mixed model ANOVA. We correlated the individual ISCs with the behavioral explicit measures using Pearson’s coefficient. We set the significance threshold at *P* < 0.05, and corrected for multiple comparisons of the explicit measures by false discovery rate (FDR) procedure (Benjamini and Hochberg 1995). For correlation and ANOVA analyses, we used SPSS (IBM SPSS Statistics for Windows 2021).

## Results

To test the first hypothesis, that is, whether the neural alignment would increase during paradoxical intervention, we started by splitting the intervention data into three equal parts (beginning, middle, end) and found the best MCCA component with the highest intersubject correlation for all timeslots separately. We focused on the delta band based on earlier MCCA research (Lankinen et al. 2014, 2018). We found the components with the strongest intersubject correlation for each intervention group, timeslot, and validation set. To now test H1, we ran a mixed model ANOVA for the three time points and three intervention groups and found a significant time^*^group interaction effect (*F*(4,234) = 3.808, *P* = 0.005). Next, we ran separate repeated measures ANOVA-s for each intervention group to see which group drives this effect and found that while there was no time-effect in inconsistency (*F*(2) = 1.25, *P* = 0.286) or control (*F*(2) = 2.03, *P* = 0.132) groups, there was a significant effect in the paradoxical thinking group (*F*(2) = 3.26, *P* = 0.039, Fig. 1c) with the neural alignment increasing as the intervention progressed. Source analysis estimated the strongest component in the only significant timeslot—the end part—to originate from precuneus (Fig. 1b). We thus find enough evidence to reject the first null hypothesis for paradoxical thinking, but not for inconsistency approach. We ran similar analyses for theta and alpha frequency bands, but neither of the mixed model ANOVA-s produced significant time^*^group effects (*F*(4,234) < 1.421, *P* > 0.216).

Since paradoxical thinking group was the only experimental group to show any change in the neural synchronization and the third timeslot there the only one to have a significant component, we conducted correlation analysis to test H2 between the third timeslot best component ISC and the T2-T1 differences in the behavioral explicit measures. We found a significant correlation between the ISC and the T2-T1 change in feeling thermometer (*R* = 0.436^*^, *p*_FDR-cor_ = 0.045, Fig. 1e)—the bigger the correlation, the warmer feelings toward vaccine-opposers participants reported at T2 compared to T1. This allows us to reject the second null-hypothesis, even though the rest of the correlations with third ISC were insignificant (*P* > 0.148, Table 1).

**Table 1:** Correlations in the paradoxical thinking group: Neural alignment End, Agreement End-Beginning and T2-T1 changes in all explicit measures

		**Neural alignment End**	**Agreement End-Beginning**	**Negativity T2-T1**	**Threat T2-T1**	**Feeling thermometer T2-T1**	**Dehumanization T2-T1**	**Warmth T2-T1**	**Competence T2-T1**	**Measures against vaccine-opposers T2-T1**	**Positive emotions T2-T1**	**Negative emotions T2-T1**
**Neural alignment End**	**R**	—										
**Agreement End-Beginning**	**R**	0,017	—									
	**p**	0,919										
**Negativity T2-T1**	**R**	−0,098	0,072	—								
	**p**	0,554	0,663									
**Threat T2-T1**	**R**	−0,142	0,246	0,177	—							
	**p**	0,387	0,131	0,280								
**Feeling thermometer T2-T1**	**R**	**.436** ^^**^^	0,221	−0,048	−0,107	—						
	**p**	**0,005**	0,176	0,771	0,518							
**Dehumanization T2-T1**	**R**	0,264	0,167	0,031	0,135	0,290	—					
	**p**	0,144	0,361	0,867	0,463	0,107						
**Warmth T2-T1**	**R**	−0,243	0,100	−0,276	0,148	−0,272	0,260	—				
	**p**	0,173	0,579	0,120	0,412	0,126	0,151					
**Competence T2-T1**	**R**	−0,200	0,184	−0,197	−0,038	−0,087	0,272	**.748** ^^**^^	—			
	**p**	0,264	0,307	0,272	0,834	0,631	0,132	**0,000**				
**Measures against vaccine-opposers T2-T1**	**R**	0,210	**.474** ^^**^^	0,172	−0,008	−0,050	0,027	−0,007	−0,004	—		
	**p**	0,240	**0,005**	0,340	0,964	0,781	0,881	0,968	0,981			
**Positive emotions T2-T1**	**R**	−0,029	−0,163	−0,164	−0,082	−0,130	0,105	**.418** ^^*^^	**.386** ^^*^^	−0,143	—	
	**p**	0,861	0,322	0,317	0,621	0,429	0,567	**0,016**	**0,027**	0,427		
**Negative emotions T2-T1**	**R**	0,066	−0,048	**−.356** ^^*^^	−0,036	−0,123	−0,255	−0,158	0,084	0,025	−0,024	—
	**p**	0,692	0,772	**0,026**	0,830	0,454	0,158	0,380	0,643	0,889	0,885	

*Note*. Significant correlations marked.

Since we found a change in neural synchronization in the paradoxical thinking group, we investigated whether it also surfaces on the behavioral level and looked at how much participants agreed with the statements in the beginning, middle, and end part of the manipulations. Mixed model ANOVA revealed a highly significant time^*^group effect for the agreement level (*F*(4,234) = 18.939, *P* < 0.001). Investigating further, we found a significant time-effect in paradoxical (*F*(2) = 49.58, *P* < 0.001, Fig. 1d) group with agreement with statements lessening over time. Also inconsistent statements had a time-effect (*F*(2) = 13.91, *P* < 0.001) but the neutral control statements did not (*F*(2) = 1.50, *P* = 0.230). We checked whether the reduction in agreement (end – beginning difference) predicted any behavioral measures for the paradoxical group and found a significant correlation with measures against vaccine-opposers (*R* = 0.474^**^, *P*_FDR-cor_ = 0.045, Fig. 1f). There were no other significant correlations (*P* > 0.130, Table 1).

## Discussion

We found that neural activation grows more synchronized between participants while undergoing the paradoxical thinking intervention. While we were unable to detect any significant intersubject correlations during the first four minutes of paradoxical thinking intervention, a significant component appeared during the last two minutes of the intervention. In all paradoxical thinking studies so far, the intervention is viewed as one unit and any measures of effect are collected after the intervention or averaged across items (Hameiri et al. 2020; Hautala et al. 2022; Knab and Steffens 2022). Even when conflict-supporting attitudes have been reported as reducing over time, the research has compared pre and post intervention measures (Hameiri et al. 2016). Hameiri et al. once tested the qualitative “sweet spot” of the necessary extremity (measured by agreement with the statements) for the mental unfreezing to occur (Hameiri et al. 2020) but the quantitative “sweet spot” had not yet been determined. We theorize our findings reflect on the necessary length or depth of the paradoxical thinking intervention to be effective in changing attitudes when applied as a one-time light touch (brief and inexpensive) intervention (Paluck et al. 2021). According to a recent thorough review (Paluck et al. 2021), 76% of prejudice reduction interventions in the last 20 years have been light touch interventions but only 8% of them measured outcomes even a day after the intervention. Not only do we measure the affected attitudes 2 days after the experiment on average, but we also relate the neural processes happening during the intervention to the self-reported outcomes.

Intersubject correlation or neural alignment is often used as a marker for shared neural activity and is especially valid in response to naturalistic and complex (real-life like) stimuli (Hasson et al. 2012; Nummenmaa et al. 2018). Using real-life complex materials is a direction that has risen to interest in social neuroscience lately (Adolphs et al. 2016; Leong et al. 2020; Katabi et al. 2023). In addition to a broad collection of fMRI intersubject correlation studies, EEG (Maffei 2020; Kaneshiro et al. 2021; Ueno and Shimada 2023) and MEG (Lankinen et al. 2016; Zhang et al. 2017; Thiede et al. 2020) have more recently been shown to be promising approaches for capturing shared neural processes, allowing for ecological validity in naturalistic situations (Levy et al. 2021b). Superior temporal resolution of MEG and EEG allow for investigating fast brain activity and cortical rhythms, possibly carrying information related to diverse functional processes of the brain.

The intersubject correlation we observed in the third part of the intervention was estimated to stem from precuneus, which is an area involved in processing others’ mental states and constructing different perspectives in affective and cognitive mentalizing (Arioli et al. 2021), and also self-related mental representations in the network of self-consciousness (Cavanna and Trimble 2006; Cavanna et al. 2008). This finding validates the theory of paradoxical thinking intervention unfreezing previously held attitudes and changing perspective (Hameiri et al. 2020) and extends the theory on the exact mechanism: critically reviewing one’s own identity and increased perspective-taking ability. Future research on paradoxical thinking intervention can benefit from these findings by adding measures of these phenomena to assess the interventional effect.

Moreover, the amount of the synchronized brain activity in precuneus predicted change in feeling thermometer: the stronger the correlation, the bigger the positive change in outgroup affect. There is a movement in social neuroscience suggesting that neural markers predict future behavior change better than self-report measures (Falk et al. 2010, 2012; Berkman and Falk 2013; Levy et al. 2022). The intervention we analyze originates from a preregistered data collection where we described a pre-post analysis (https://osf.io/uwmpa/?view_only=e48e1c57ad8f4639ba35a974b92122aa). In the pre-post analysis, we find modulations of negative attitudes against the outgroup but not in the feeling thermometer (A. Kluge and J. Levy, unpublished observations, https://osf.io/preprints/psyarxiv/w65pm). Now, by finding the neural marker for the interventional effect, we demonstrated that it actually predicts change in outgroup affect, previously unnoticed due to intersubject variability. We show that analyzing the intervention data uncovers neural mechanisms, that the pre-post analysis (especially on self-report level) fails to see, that well predict future behavior change.

We argue that neuroimaging has two main benefits for intervention research. First, neural data is richer and more sensitive than behavioral and can precisely pinpoint the mechanisms that the intervention influences. Second, the neural markers are provenly better predictors of real-life attitude change than behavioral self-reports (Falk et al. 2010, 2012; Berkman and Falk 2013; Gabrieli et al. 2015; Hautala et al. 2022; Levy et al. 2022). The present study pioneers with a new approach to the study of interventions: it extends the recent strategy of using neuroimaging to improve the evaluation of interventions’ impact and implements a radically new tactic—a time-resolved monitoring of the way that interventional stimuli may alter neural activity underlying mental processes. By recording the neural dynamics during the course of interventions and testing whether such alterations may cause subsequent modulations in the processing of intergroup affect and self-reported attitudes, this neuroscientific tactic can reveal mechanisms that would help in adding or dropping parts of the interventions on the way to make it more effective. This pioneering approach would guide scholars and practitioners in designing and improving interventions, thereby tackling a critically pressing challenge in the field of intergroup interventions (Paluck et al. 2021). Such outcome would not only contribute to science but also to society’s integrity, diversity, and wellbeing.

Finally, we also observed a significant modulation in the agreement with the intervention statements: the agreement significantly decreased over time in the paradoxical and in the inconsistent condition. Further, the change in the agreement in the paradoxical condition predicted the change in support of measures against vaccine-hesitancy: the more the agreement decreased, the less people supported discriminative measures after the experiment compared to baseline. The agreement with the statements has previously been shown to be an important factor for the “unfreezing” effect of the intervention (Hameiri et al. 2020), and predict a suppression of neural outgroup bias (Hautala et al. 2022). We show that the agreement is not constant during the intervention and instead, decreases over time. We speculate that the build-up we see in the disagreement with statements is related to the build-up of the neural alignment, since both seem to happen halfway through the experiment (the change in agreement earlier than in neural alignment). However, we cannot see a direct correlation—this might be due to the analysis design. We divided the data into three blocks to optimize the analysis considering the paradigm length, and thus cannot evaluate more specifically what happens during the middle block. In future studies, this research could be repeated with multiple versions of statements that would enable to examine whether some specific stimuli are more effective in triggering neural alignment.

## Data Availability

As MEG data cannot be fully anonymized, it cannot be made publicly available by Finnish data protection laws. The individual behavioral data cannot be shared following the ethics permit behind this submission and GDPR.
